# Off-season survival and life history of beet armyworm, *Spodoptera exigua* (Hubner) on various host plants

**DOI:** 10.1038/s41598-024-64639-8

**Published:** 2024-06-14

**Authors:** L. Rajesh Chowdary, G. V. Suneel Kumar, S. Bharathi, O. Sarada, Yalavarthi Nagaraju, Keerthi Manikyanahalli Chandrashekara, Giri Naga Harish

**Affiliations:** 1 Agricultural Research Station, Acharya N. G. Ranga Agricultural University, Darsi, Prakasam, 523247 India; 2 Administrative Office, Acharya N. G. Ranga Agricultural University, Lam, Guntur, 522034 Andhra Pradesh India; 3 Regional Agricultural Research Station, Acharya N. G. Ranga Agricultural University, Lam, Guntur, 522034 Andhra Pradesh India; 4 Central Sericultural Research and Training Institute, Central Silk Board, Berhampore, West Bengal India; 5 Division of Crop Protection, ICAR-Indian Institute of Horticultural Research, Bengaluru, 560 089 India

**Keywords:** Demographic parameters, Development period, Growth index, Host plants, Life cycle, Off-season survival, *Spodoptera exigua*, Entomology, Ecology, Zoology

## Abstract

The beet armyworm, *Spodoptera exigua* (Hubner) (Lepidoptera: Noctuidae), has become a significant pest of chickpea in recent years. The polyphagous nature allows it to survive on various hosts during the off-season, creating a great menace to the crop in the following season. To assess the incidence and document the alternate hosts of *S. exigua*, a rapid roving survey was conducted in 11 chickpea-growing areas of Prakasam district, Andhra Pradesh, India. Additionally, the life history traits of *S. exigua* were studied on major alternate host plants under laboratory conditions (27 ± 1 °C and 70 ± 2% RH) to understand the survival, life expectancy and potential contribution to future populations. The results show that, among the different crops surveyed, the maximum larval incidence was noticed in maize (1.93 larvae/plant), cowpea (1.73 larvae/plant), and sunflower (1.68 larvae/plant) during the off-season. Life history studies of *S. exigua* showed that highest larval survival percentage was observed on chickpea (83.6%), while the lowest was on maize (44.5%). The mean developmental time for larvae was longest on maize (27.1 days) and shortest on chickpea (14.9 days). Larvae did not develop beyond the third instar when fed with chilli. The growth index statistics showed chickpea (9.2) was the most suitable host plant, whereas maize (0.9) was the least suitable host. The age-stage-specific survival rate (S_xj_) varied across developmental stages, and the survival curves overlapped, indicating different growth rates among individuals. The life expectancy (e_xj_) at age zero was highest on groundnut (37.06 days). The intrinsic rate of increase (r) of *S. exigua* was lowest on maize (0.10 ± 0.0013) and highest on chickpea (0.22 ± 0.0010). Similarly, the net reproductive rate (R_0_) was highest on chickpea (846.39 ± 18.22) and lowest on maize (59.50 ± 2.06). The population doubled every 3.08 ± 0.011 days on chickpea compared to 7.22 ± 0.80 days on maize. The study conclusively indicates that chickpea and sunflower, primarily cultivated during the rabi season in India, are the most preferred hosts for *S. exigua*. In contrast, maize and cotton, mainly grown during the kharif season, are less preferred and merely support the pest's survival. Consequently, *S. exigua* switches hosts between different crops growing seasons, so effective management of *S. exigua* during the kharif season can help prevent pest outbreaks during the rabi season.

## Introduction

The beet armyworm, *Spodoptera exigua* (Hubner) (Lepidoptera: Noctuidae), is recognized as a highly polyphagous pest and cause potential damage to a wide range of economically important crops in tropical and subtropical climates^1^. The *S. exigua* observed feeding on 170 species, belonging to 35 families of plants and causes significant damage^1–3^. In India, the beet armyworm inflicts damage on a wide range of crops such as cotton, castor bean, spinach, tomato, cabbage, chillies, and alfalfa. Occasionally, outbreaks of this pest lead to substantial economic losses in various economically important crops across India^4–7^. Availability of host plants of both highly suitable and less suitable or non-suitable host plants can influence the *S. exigua* spatio-temporal population dynamics, density, spread, and distribution pattern in the agricultural landscape^7,8^. Further, off-season adaptability to various hosts and its capacity to develop resistance to insecticides, which play an important role in its outbreaks, make its management difficult^3^.

Studies focused on the influence of the host on the developmental biology of insect pests provide the basis for understanding the suitability of the host and the potential extent of damage that they can cause and can provide inputs for effective pest management strategy^9–12^. Factors such as shorter developmental time, lower mortality rates, and enhanced reproductive capacity serves as essential biological markers to determine the adaptability of insects to specific host plants^3^. For instance, *S. exigua* has been found to have better growth and development on cauliflower compared to peas and wheat. On the other hand, peas and wheat were found to be unsuitable for *S. exigua*, suggesting that these plants could potentially be used as trap crops^9^. Host plant characteristics affect the life-history traits of herbivorous insects. Host plant morphological, biochemical, and nutritional properties can influence the life cycle, fecundity, survival, longevity, body size, and development of an insect population^3,9,10^. The *S. exigua* can sustain their populations by transitioning between different hosts such as vegetable crops, field crops and weed hosts, and thereby maintaining its population throughout the year^8,13,14^. For instance, in Brazil, it was noted that it survived on the cover crops such as millets during the offseason, resulted in increased infestation rates of *S. exigua* on cotton in the following season^15^. Therefore, understanding the relationship between *S. exigua* and its host plants and the biological, ecological susceptibilities of *S. exigua* could provide valuable insights for researchers aiming to devise the effective management strategies against this pest^16^.

The biology of insects is closely linked to the availability of suitable hosts, suggesting that the essential nutritional components and quality of host plants significantly influence the life history parameters and reproductive fitness of insects^3,17,18^. The presence of immature stages on any crop does not necessarily guarantee that the plant is acting as suitable host for *S. exigua*. To confirm whether a plant is a suitable host for a specific insect pest, it must be capable of completing its life cycle on the host plant. Understanding these factors and the resistance-susceptibility traits of different crops to *S. exigua* will assist growers in implementing appropriate control measures. Moreover, comprehending the biological, reproductive, and population parameters of any insect pest is essential for devising effective and sustainable pest management strategies. Comparing life table factors is a valuable technique for examining the influence of host plants on the fitness of insect pests. Therefore, we propose that studying the biology and constructing life tables for off-season hosts can provide fundamental insights into off-season survival, future population contribution, and the development of Integrated Crop Management (ICM) practices.

## Results

### Incidence of *S. exigua* on different crops and weeds

The results of the field survey conducted in Prakasam district of Andhra Pradesh, show that the larvae of *S. exigua* were found to feed on 15 plant species, including two weed species. The off-season survey data revealed that the survival of *S. exigua* larvae varied between 0.5 to 1.93 larvae per plant, while leaf damage ranged from 3.94 to 37.92% (Table 1). During the off-season of 2016–2017, significant variations were observed in the incidence of *S. exigua* on various crops, including pulses (such as greengram, blackgram, cowpea, and soybean), oilseeds (groundnut, castor, and sunflower), commercial crops (chilli and cotton), vegetables (onion and brinjal), other crops (maize and tobacco nurseries), and weeds (wild amaranthus and *Euphorbia geniculata*) across the 11 mandals surveyed. Among the different crops examined, the highest larval incidence was observed on maize (1.93 larvae per plant), cowpea (1.73 larvae per plant), sunflower (1.68 larvae per plant), and groundnut (1.47 larvae per plant) during the off-season of chickpea cultivation (both pre and post *Rabi* season). In contrast, minimal larval populations were found on the alternate weed hosts *E. geniculata* and wild Amaranthus, with only 0.22 and 0.24 larvae per plant, respectively. Sunflower exhibited the highest percentage of leaf damage (37.92%), followed by cowpea (34.41%), groundnut (32.72%), and tobacco (30.13%), while *E. geniculata* showed the lowest damage at 3.94%. Similarly, the visual leaf damage rating was highest for sunflower (4.69) and lowest for *E. geniculata* (1.29).

**Table 1:** Off-season survival of *Spodoptera exigua* and the extent of damage in different crops in the Prakasam district.

Month of survey	Mandal	Name of the crop and variety	Crop stage	No. of larvae/plant*	% Leaf damage**	Leaf damage rating^#^
August, 2016	Pedaaraveedu	Onion	Bulb formation	1.11 (1.05)^f^	25.25 (30.09)^d^	3.42 (1.85)^e^
August, 2016	Darsi	Greengram, ML 267	Pod formation	0.87 (0.93)^g^	20.39 (26.79)^e^	2.94 (1.72)^f^
September, 2016	Markapur	Chilli, Indam 5	Vegetative	1.17 (1.06)^ef^	18.34 (25.27)^e^	2.73 (1.65)^f^
September, 2016	Chirala	Groundnut TAG 24	Peg formation	1.47 (1.21)^cd^	32.72 (34.77)^bc^	4.17 (2.04)^bc^
September, 2016	Cumbum	Tobacco nursery	Nursery	1.33 (1.14)^de^	30.13 (33.22)^c^	3.91 (1.98)^cd^
September, 2016	Kurichedu	Castor PCH 111	Capsule formation	1.05 (1.02)^f^	20.14 (26.63)^e^	2.91 (1.71)^f^
October, 2016	Martur	Cotton, NDLH 1938	Boll formation	0.74 (0.86)^gh^	8.15 (16.34)^g^	1.71 (1.30)^h^
October, 2016	Markapur	Cow pea, Meghana	Vegetative	1.73 (1.30)^ab^	34.41 (35.76)^ab^	4.34 (2.07)^ab^
October, 2016	J. Panguluru	Black gram, LBG 752	Flowering	0.80 (0.89)^gh^	12.43 (20.54)^f^	2.14 (1.46)^g^
November, 2016	Darsi	Soy bean, JS 335	Vegetative	0.65 (0.80)^h^	8.27 (16.27)^g^	1.73 (1.31)^h^
November, 2016	Addanki	Maize, 30V92	Seedling	1.93 (1.39)^a^	28.93 (32.36)^cd^	3.79 (1.94)^de^
March, 2017	Darsi	Pig weed, *Amaranthus* spp	Flowering	0.24 (0.47)^j^	6.34 (14.36)^g^	1.53 (1.23)^h^
March, 2017	Talluru	Brinjal, Mahyco hybrid	Vegetative	0.50 (0.71)^i^	12.77 (20.67)^f^	2.18 (1.47)^g^
March, 2017	Mundlamuru	Sunflower, private hybrid	Heading	1.68 (1.29)^bc^	37.92 (37.96)^a^	4.69 (2.17)^a^
April, 2017	Darsi	*Euphorbia geniculata*	Flowering	0.22 (0.44)^j^	3.94 (11.20)^h^	1.29 (1.13)^i^
LSD (P = 0.05)	–	–	–	0.09	2.46	0.10
CV%	–	–	–	11.6	13.48	7.93
Fp	–	–	–	0.4	0.1	0.1

^#^Damage rating (1 ≤ 10% leaf area damaged and 9 ≥ 80% leaf area damaged).

*Figures in parenthesis are square root transformed values.

**Figures in parenthesis are arc sine transformed values.

In a column, means followed by a common letter (s) are not significantly different by DMRT (P = 0.05).

### Life history of *S. exigua* on different host plants

The results of the development period (Table 2), adult longevity (Table 3), and life span of *S. exigua* (Table 4) reared on different host plants are presented here. The duration of the egg incubation period differed among the host plants, with cotton exhibiting the longest period (3.5 days), whereas chilli showed the shortest incubation period (2.7 days) (Table 2). Host plants significantly influenced the percentage of egg hatch, with the highest observed on chickpea (92.7%) and the lowest on chilli (82.3%). Egg hatch rates on chickpea were comparable to those on groundnut (91.3%) and maize (90.2%). Significant variations in the duration of *S. exigua* larval instars were noted when reared on different host plants (Table 2). Overall, larvae exhibited faster development from the first to sixth instars when fed on chickpea (14.9 days). Chilli impeded larval development and growth, as evidenced by larvae failing to progress beyond the third instar and displaying prolonged developmental times during the first and second instars compared to other host plants studied. Regarding larval survival, the larvae reared on chickpea displayed the highest percentage (83.6%), while maize exhibited the lowest (44.5%). Notably, larvae did not survive on chilli (0%) under laboratory conditions, despite 82.3% of eggs successfully hatching into first instar larvae. There was a notable difference in the pupal period among the host plants. When larvae were fed on chickpea, the pupal duration of *S. exigua* was only 7.8 days, while larvae fed on maize showed a significantly longer pupal duration of 11.3 days (Table 2). Similar durations of the pupal stage were recorded in blackgram (8.7 days) and greengram (8.9 days).

**Table 2:** Longevity of immature stages of *Spodoptera exigua* reared on different host plants.

Host plants	Mean (± SE) of development stages of *S. exigua*			
	Incubation period (days)	Larval period (days)	Pupal period (days)	Pupal weight (mg)
Chickpea	3.1 ± 0.1^bc^	14.9 ± 0.5^g^	7.8 ± 0.2^g^	89.6 ± 0.8^a^
Sunflower	3.0 ± 0.1^cd^	16.0 ± 0.4^f^	9.7 ± 0.1^c^	85.9 ± 0.9^b^
Castor	3.0 ± 0.1^cd^	16.8 ± 0.3^ef^	9.4 ± 0.1^cd^	87.5 ± 0.7^ab^
Cotton	3.5 ± 0.1^a^	24.7 ± 0.3^b^	9.2 ± 0.2^de^	55.7 ± 0.8^d^
Blackgram	3.3 ± 0.1^abc^	17.5 ± 0.3^e^	8.7 ± 0.2^f^	77.6 ± 1.0^c^
Greengram	3.4 ± 0.2^ab^	21.8 ± 0.3^c^	8.9 ± 0.1^ef^	47.1 ± 1.0^e^
Maize	3.2 ± 0.1^abc^	27.1 ± 0.4^a^	11.3 ± 0.2^a^	30.8 ± 0.6^f^
Groundnut	3.0 ± 0.1^cd^	18.9 ± 0.4^d^	10.2 ± 0.2^b^	56.9 ± 0.8^d^
Chilli	2.7 ± 0.1^d^	–	–	–

Means accompanied by the same letter in the same rows are not significantly different at P < 0.05 based on DMRT.

**Table 3:** Longevity of adult stage of *Spodoptera exigua* reared on different host plants.

Host plants	Mean (± SE) of development stages of *S. exigua*			
	Male longevity (days)	Female longevity (days)	Oviposition period (days)	Fecundity (no.)
Chickpea	9.2 ± 0.1^b^	10.1 ± 0.1^c^	7.7 ± 0.2^c^	2066.1 ± 15.2^a^
Sunflower	6.5 ± 0.3^de^	7.9 ± 0.2^e^	6.6 ± 0.1^d^	1282.83 ± 9.6^f^
Castor	8.6 ± 0.2^c^	11.3 ± 0.2^b^	9.0 ± 0.1^a^	1919.5 ± 12.2^b^
Cotton	6.0 ± 0.3^ef^	8.3 ± 0.2^e^	4.4 ± 0.1^e^	465.2 ± 3.8^g^
Blackgram	9.3 ± 0.1^b^	12.3 ± 0.2^a^	8.6 ± 0.4^ab^	1749.2 ± 16.6^d^
Greengram	7.1 ± 0.3^d^	9.4 ± 0.1^d^	6.9 ± 0.2^d^	1397.3 ± 11.9^e^
Maize	5.9 ± 0.2^f^	7.1 ± 0.2^f^	3.3 ± 0.1^f^	286.5 ± 3.82^h^
Groundnut	9.9 ± 0.1^a^	11.0 ± 0.2^b^	8.2 ± 0.3^bc^	1862.62 ± 15.9^c^
Chilli	–	–	–	–

Means accompanied by the same letter in the same rows are not significantly different at P < 0.05 based on DMRT.

Males emerged first, and a day later, females emerged.

Egg laying started on 2nd day after the release.

**Table 4:** Influence of different host plants on the development of *Spodoptera exigua.*

Host plants	Mean (± SE) of development stages of *S. exigua*		
	Pre-imaginal developmental period (days)	Total life cycle period (days)	Overall survival (%)
Chickpea	25.7 ± 0.5^f^	35.3 ± 0.5^e^	70.9 ± 1.1^a^
Sunflower	28.6 ± 0.4^e^	35.8 ± 0.4^e^	58.5 ± 1.0^c^
Castor	29.1 ± 0.3^e^	39.1 ± 0.4^d^	64.3 ± 1.1^b^
Cotton	37.4 ± 0.4^b^	44.5 ± 0.5^b^	41.2 ± 1.0^d^
Blackgram	29.5 ± 0.4^e^	40.2 ± 0.5^d^	39.1 ± 0.9^d^
Greengram	34.1 ± 0.3^c^	42.3 ± 0.4^c^	23.8 ± 0.6^f^
Maize	41.0 ± 0.5^a^	47.5 ± 0.5^a^	9.9 ± 0.2^g^
Groundnut	32.1 ± 0.5^d^	42.5 ± 0.5^c^	34.6 ± 1.6^e^
Chilli	–	–	–

Means accompanied by the same letter in the same rows are not significantly different at P < 0.05 based on DMRT.

There were notable differences in the pupal survival (adult eclosion) of *S. exigua* across various host plants. The survival rate on chickpea (84.8%) was significantly higher (P < 0.05) compared to other hosts. The percentage of individuals successfully reaching adulthood from the pupal stage was relatively low on blackgram (54%) and groundnut (48.5%). Conversely, the lowest pupal survival percentage was recorded on maize (22.2%). The heaviest pupae (89.6 mg) were observed on chickpea, while the lightest pupae (30.8 mg) were found on maize (Table 2). Statistically significant differences (*P* < 0.05) in longevity were observed between male and female insects depending on the host plant. Male adults reared on groundnut (9.9 d) displayed significantly longer lifespan, as did females reared on blackgram with the most extended adult longevity (12.3 days) (Table 3). The longest oviposition period (9.0 days) was observed on castor, while the shortest was noted on maize (3.3 days). The highest fecundity was observed when the larvae were fed with chickpea, which had the highest number of total eggs laid per female during the larval stage (258.2 eggs per female), while the lowest number of eggs laid was observed on maize (94.3 eggs per female).

The pre-imaginal development period (41 days) and total lifespan (47.5 days) were significantly longer on maize (Table 4). However, the shortest pre-imaginal development period and total lifespan of the beet armyworm were on chickpea (25.7 and 35.3 days) and sunflower (28.6 and 35.8 days), respectively. Different host plants affected the survival of *S. exigua* till adulthood (P < 0.05), with the highest percent survival recorded when the larvae were reared on chickpea (70.9%) and the lowest on maize (9.9%) (Table 4). Table 5 displays the growth index and mortality percentages of larvae and pupae of *S. exigua* on various hosts. Chickpea demonstrated the highest growth indices for larvae and pupae, with values of 5.7 and 9.2, respectively. In contrast, the growth index of larvae and pupae on maize was 1.7 and 0.9, respectively, the lowest among all hosts.

**Table 5:** The growth index (GI) and mortality of immature stages of *Spodoptera exigua* on different host plants.

Host plants	Larva		Pupa	
	Growth index	% Mortality	Growth index	% Mortality
Chickpea	5.7^a^	16.4 ± 0.7^g^	9.2^a^	15.3 ± 1.2^g^
Sunflower	4.7^b^	26.1 ± 0.7^e^	6.1^c^	20.8 ± 1.4^ef^
Castor	4.7^b^	20.9 ± 0.9^f^	6.9^b^	18.8 ± 0.8^f^
Cotton	2.2^f^	46.6 ± 0.8^b^	4.5^d^	22.9 ± 1.4^e^
Blackgram	4.2^c^	27.6 ± 0.8^de^	4.5^d^	46.1 ± 1.2^d^
Greengram	2.9^e^	37.5 ± 0.7^c^	2.7^f^	62.0 ± 0.9^b^
Maize	1.7^g^	55.5 ± 0.6^a^	0.9^g^	77.8 ± 0.3^a^
Groundnut	3.8^d^	28.6 ± 0.9^d^	3.4^e^	51.6 ± 2.0^c^

Means followed by different letters in the same columns are significantly different (P < 0.05) by DMRT.

### Survival rate, life expectancy and reproductive value of *S. exigua* on different hosts

**Figure 1:**
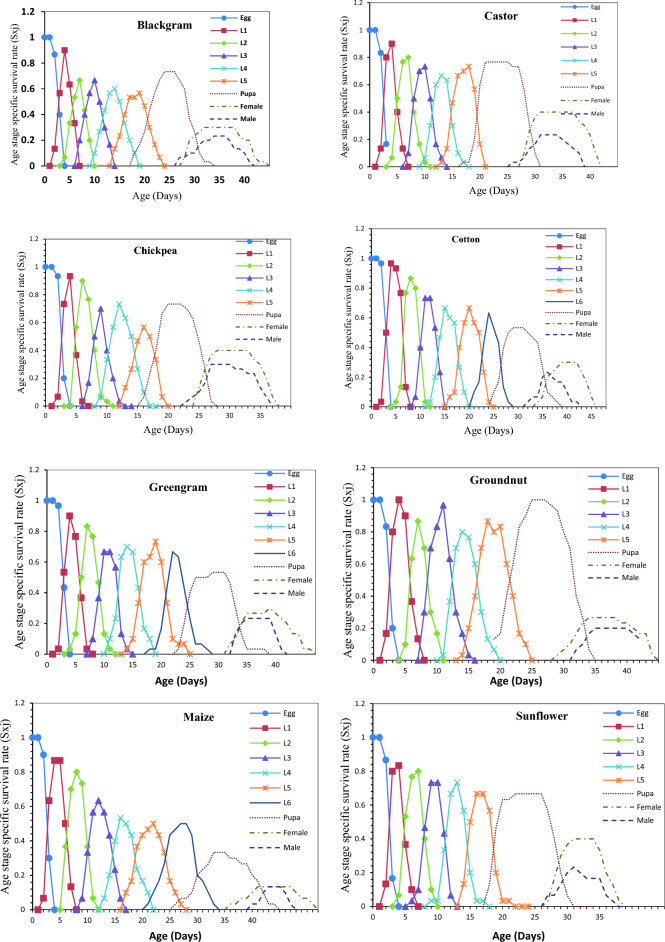
Age-stage-specific survival rate (S_xj_) of *Spodoptera exigua* on different host plants.

**Figure 2:**
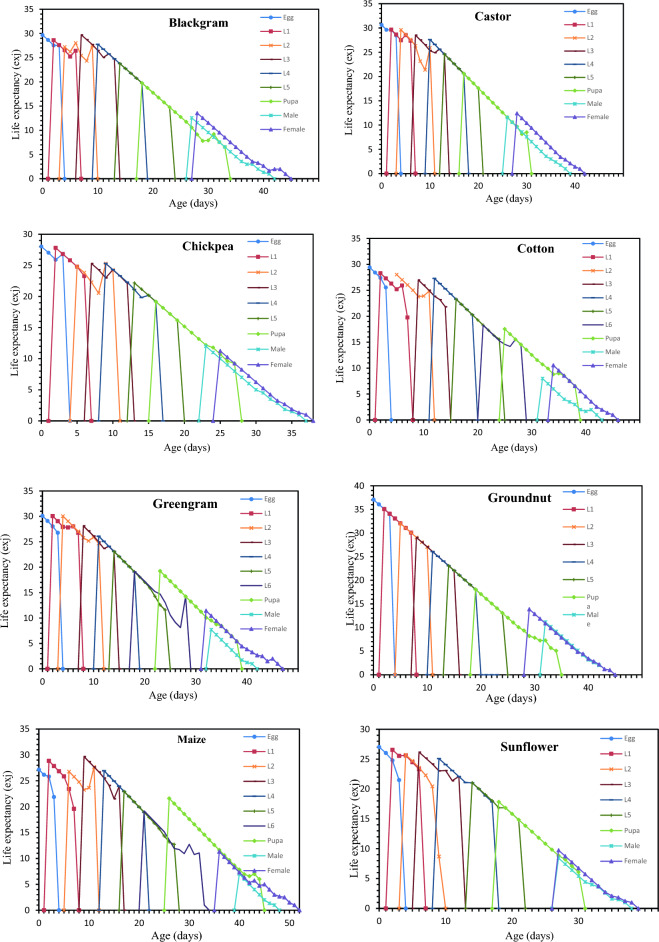
Age-stage life expectancy (e_xj_) of *Spodoptera exigua* on different host plants.

**Figure 3:**
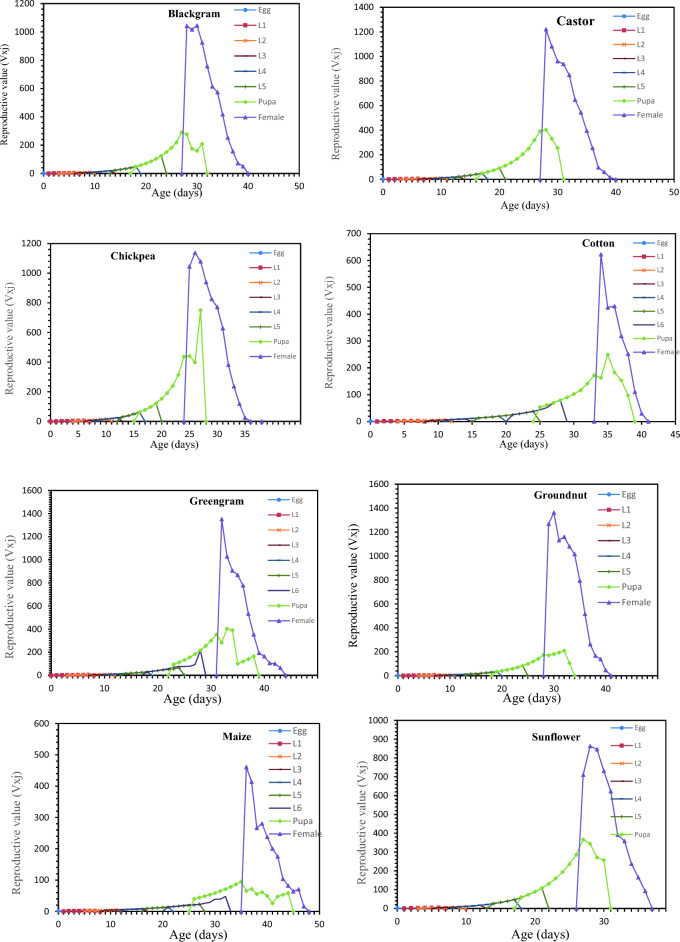
Age-stage reproductive value (V_xj_) of *Spodoptera exigua* on different host plants.

**Figure 4:**
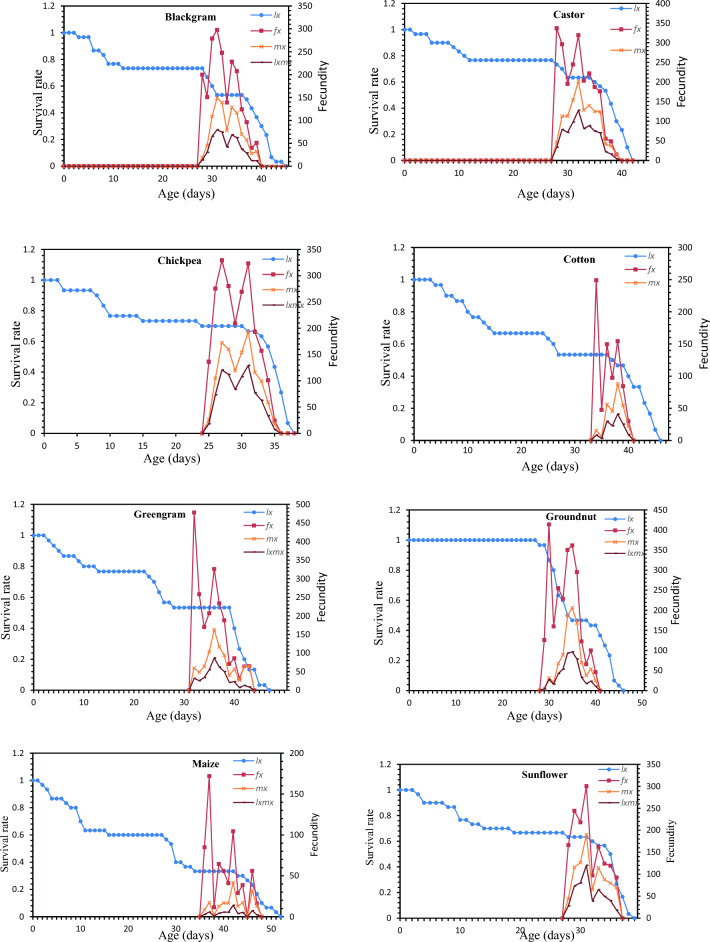
The age-specific survival rate (lx), female age-specific fecundity (fx), age-specific fecundity (mx), and age-specific maternity (lx.mx) of *Spodoptera exigua* on different host plants.

**Figure 5:**
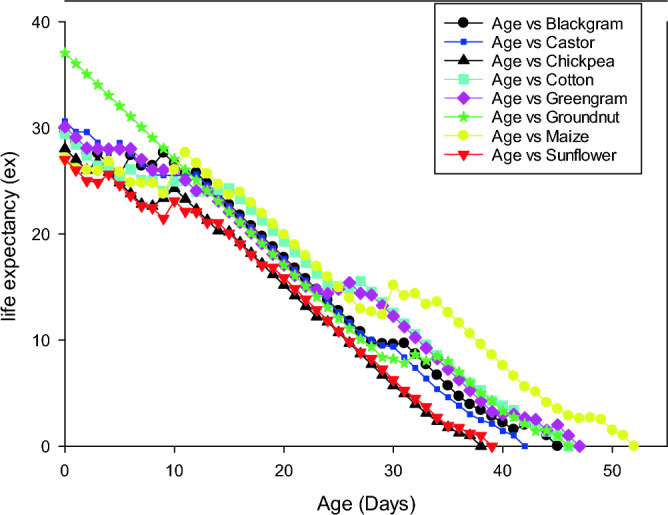
Age-specific life expectancy (e_x_) of *Spodoptera exigua* on different host plants.

**Figure 6:**
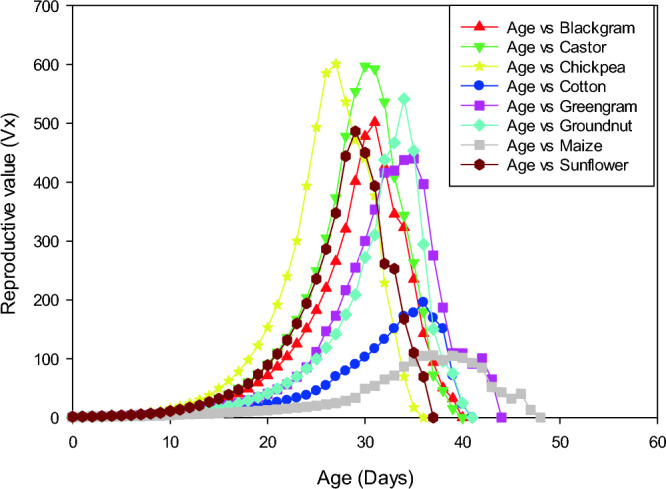
Reproductive value (v_x_) of *Spodoptera exigua* on different host plants.

Age-stage-specific survival rate (S_xj_) shows the probability that a newly laid egg of *S. exigua* survive to each age-stage unit (Fig. 1). The figure represents survivor curve and stage differentiation among individuals of *S. exigua* reared on different hosts. The rearing of *S. exigua* on different hosts had a significant effect on the probability that a newly laid egg of *S. exigua* will survive to the adult stage. The values varied across developmental stages, and the survival curves were overlapped, which can be attributed to the fact that different individuals grow at different rates. The age-stage-specific life expectancy (e_xj_) describes an expected lifespan of *S. exigua* individuals of age x and stage j on eight different hosts. The value of *e*_*xj*_ showed a downward trend on all the hosts studied under the laboratory conditions, which indicate the life expectancy of the individual was reduced with the advancement of age (Fig. 2). The reproductive value (*V*_*xj*_) of the *S. exigua*, indicates the contribution of a *S. exigua* individuals at age x and stage j to the future populations presented in Fig. 3.The age-specific survival rate (l_x_), fecundity (m_x_), and maternity (l_x_.m_x_) values of *S. exigua* are shown in Fig. 4. The curve of l_x_ is the pooled and simplified survival rate of S_xj_ curves of different stages in Fig. 1, and it represents the probability that a newly laid egg will survive to age x. Similarly, age-specific life expectancy (e_x_) and reproductive value (v_x_) of *S. exigua* on different host plants was presented in the Figs. 5 and 6, respectively.

### Population growth parameters of *S. exigua* (mean ± SE) on eight different host plants

Table 6 presents the intrinsic rate of increase (r), finite rate of increase (lambda), net reproductive rate (R_0_), gross reproduction rate (GRR), mean generation time (T), and doubling time (DT) of *S. exigua* on various crops. Among the crops studied, chickpea exhibited the highest intrinsic rate of increase (0.22 ± 0.0010), finite rate of increase (1.25 ± 0.0009), net reproductive rate (846.39 ± 18.22), and gross reproduction rate (1231.09 ± 21.31), indicating strong reproductive capacity of *S. exigua* on these hosts. Maize, on the other hand, showed the lowest intrinsic rate of increase (0.10 ± 0.0013), finite rate of increase (1.10 ± 0.0001), net reproductive rate (59.50 ± 2.06), and gross reproduction rate (193.57 ± 5.28), suggesting comparatively lower reproductive efficiency. The mean generation time ranged from 29.88 ± 0.031 for chickpea to 42.16 ± 0.129 for maize, indicating the time taken for each generation to replace the previous one. Chickpea also displayed the shortest doubling time (3.08 ± 0.011), while maize exhibited the longest doubling time (7.22 ± 0.80), reflecting their respective population growth rates. These results provide insights into the reproductive biology and population dynamics of these agricultural crops.

**Table 6:** Population growth parameters of *Spodoptera exigua* (mean ± SE) on eight different host plants.

Host	Intrinsic rate of increase ®	Finite rate of increase (lambda)	Net reproductive rate (R_0_)	Gross reproduction rate (GRR)	Mean generation time (T)	Doubling time (DT)
Black gram	0.18 ± 0.0010^bc^	1.21 ± 0.0013^bc^	522.61 ± 15.97^c^	971.02 ± 22.58^b^	33.26 ± 0.061^d^	4.68 ± 0.022^bc^
Castor	0.20 ± 0.0008^ab^	1.22 ± 0.0010^ab^	755.83 ± 17.89^b^	1222.32 ± 23.09^a^	32.88 ± 0.04^d^	4.43 ± 0.015^bc^
Chickpea	0.22 ± 0.0010^a^	1.25 ± 0.0009^a^	846.39 ± 18.22^a^	1231.09 ± 21.31^a^	29.88 ± 0.031^e^	3.08 ± 0.011^c^
Cotton	0.13 ± 0.0007^de^	1.14 ± 0.0001^d^	139.85 ± 3.71^e^	292.61 ± 5.69^e^	38.26 ± 0.058^b^	5.37 ± 0.032^b^
Greengram	0.16 ± 0.0009^cd^	1.18 ± 0.0012^c^	442.17 ± 13.33^d^	907.44 ± 22.68^cd^	36.88 ± 0.068^b^	4.23 ± 0.028^bc^
Groundnut	0.18 ± 0.0012^bc^	1.19 ± 0.0011^bc^	487.49 ± 13.73^c^	952.92 ± 20.87^bc^	35.09 ± 0.063^c^	3.92 ± 0.021^bc^
Maize	0.10 ± 0.0013^e^	1.10 ± 0.0001^e^	59.50 ± 2.06^f^	193.57 ± 5.28^f^	42.16 ± 0.129^a^	7.22 ± 0.80^a^
Sunflower	0.19 ± 0.0007^abc^	1.21 ± 0.0009^bc^	494.21 ± 10.92^c^	866.96 ± 14.25^d^	32.09 ± 0.039^d^	3.56 ± 0.015^c^
*P* value	< 0.0001	< 0.0001	< 0.0001	< 0.0001	< 0.0001	< 0.004

Means followed by different letters in the same column are significantly different. Standard errors were estimated using 100,000 bootstrap resampling.

## Discussion

Various characteristics of host plants, such as their quality, can impact the life cycle of herbivores, influencing factors like longevity, fecundity, and survival rates^9^. The presence of diverse host plants plays a crucial role in the population explosions of insects that feed on multiple plant species^8,13^. Research focusing on the biology and establishment of life tables is essential for predicting future population trends and developing effective management strategies^19,20^. We conducted biological studies using detached leaves commonly employed in laboratory feeding experiments to provide accurate data. This approach helps reduce variability between individual plants and ensures a fair assessment of insect parameters such as consumption and development rates across different host plant species. Our findings, consistent with previous studies utilizing whole plants, suggest no significant disparities in the population dynamics and growth parameters of *S. exigua*^21,22^. Consequently, our results are likely to reflect the host utilization patterns of *S. exigua* accurately, comparable to observations in natural field settings.

The average egg incubation period of *S. exigua* ranged from 2.7 to 3.5 days across different host plants. Previous investigations on *S. exigua* raised on synthetic diets indicated an egg incubation span of 2.5 to 5.0 days^23^. However, when reared on various host plants, the egg incubation period lasted between 2.4 to 3.7 days^2,24–26^. Findings unveiled that chickpea larvae exhibited the shortest development period (14.9 days), whereas those consuming castor and maize had notably lengthier durations (24 and 27.1 days, respectively). Similar outcomes were observed in cotton^25,26^ and maize^2^, where larval development time ranged from 10 to 29.5 days. Results from this study highlight significant variations in the pupal period among host plants, with larvae reared on maize displaying the most extended pupal duration. This finding aligns with previous research^27,28^, which noted considerable diversity in pupal durations. The nutritional quality provided by a host crop, particularly during the larval stage, primarily influences the biological parameters of any insect population^16^.

The host plant influenced the survival rate of *S. exigua* during the pupal stage, with the highest survival rate noted on chickpea (84.8%) and the lowest on maize (22.2%). Interestingly, our results indicated that *S. exigua* survived on all hosts except chilli. The differential survival rates could be attributed to variations in nutrient and biochemical constituents, including primary and secondary metabolites, among crops. These variances may affect the developmental parameters and fitness of *S. exigua* by altering digestibility and essential nutrient composition^22^. For instance, research by Meade and Hare^29^ found survival rates of *S. exigua* until pupation ranging from 40 to 100% on chrysanthemum and from 27.5 to 82.5% on celery^9^. Differential survival rates reflect the susceptibility of cultivars to pest infestation^29^. The pupal weights of *S. exigua* were highest on chickpea (89.6 mg) and lowest on maize (30.8 mg). Farahani et al.^30^ reported a similar range of pupal weights (73 to 88.1 mg) for *S. exigua* females on different soybean varieties, consistent with our findings^13^. Abdullah et al.^24^ also observed a mean pupal weight of 78.70 mg when rearing *S. exigua* on an artificial diet.

The larval and pupal stages of *S. exigua* exhibited the highest growth index on chickpea (5.7 and 9.2, respectively), accompanied by the lowest larval (16.4) and pupal (15.3) mortality rates. This indicates that chickpea is the most favorable host plant for *S. exigua*. It provides essential nutrients conducive to their growth and development, resulting in shorter developmental periods and increased survival rates. Setamou et al.^31^ emphasized that food quality can be accessed through the growth index (GI), which considers survival rate and development time. Greenberg et al.^21^ observed that higher survival rates and shorter development times correlated with elevated growth index values, indicating superior food quality. Conversely, on maize hybrid DHM117, the larval (1.7) and pupal (0.9) stages of *S. exigua* experienced the highest percentage of mortality and lower growth index values, suggesting inferior food quality. Generally, lepidopteran larvae consuming highly nutritious food demonstrate faster growth rates and complete development than those feeding on low-nutrient food sources^17^. This study ranks the host crops for *S. exigua* growth and development: as chickpea, castor, sunflower, blackgram, cotton, groundnut, greengram, and maize. Chilli was identified as the least suitable host for *S. exigua*, inhibiting their development and survival beyond the third instar larval stage. Variations in the nutritional quality of the host plants utilized by *S. exigua* larvae could account for the observed differences.

In our study, we observed notable discrepancies in the adult lifespan of *S. exigua* depending on the host plants, with females generally exhibiting longer lifespan than males, mainly when reared on groundnut, blackgram, and chickpea instead of castor. These findings align with prior research by Khalid Ahmed et al.^27^ and Saeed et al.^8,9^. Farahani et al.^26^ also documented prolonged female longevity on *Z. mays* and *G. hirsutum*. While it is commonly observed that females tend to outlive males in many species, there are inconsistencies across studies. For instance, some studies indicate that male *S. exigua* may have longer lifespan than females, while others find no significant sex-based differences in longevity^25,28,32^. This variability underscores the complexity of *S. exigua*'s biology and highlights the necessity for further research to understand the factors influencing its development and survival comprehensively. The fecundity of *S. exigua* varied significantly among the different host plants tested; more eggs were laid when raised on chickpea (2066.1 ± 15.2 eggs/female), while the fewest eggs were laid on maize (286.5 ± 3.82 eggs/female), representing a substantial difference of 1700 eggs. Similar fecundity values on major crops were reported by several researchers^24,33^; however, lower fecundity values were recorded on off-season or alternate hosts^25^. The nutritional quality significantly influences the egg-laying capacity of female insects^16^. Consistent with the 'mother knows best' hypothesis, it could be speculated that female moths of *S. exigua* exhibit selective preferences for laying eggs on hosts that offer enhanced nutritional benefits to their offspring. However, in some cases, female moths may choose host plants that do not necessarily ensure optimal fitness for their offspring^34^.

The higher intrinsic rate of increase (r_m_) observed in chickpea (0.22 ± 0.0010) was attributed to accelerated development (leading to a shorter generation time), enhanced survivorship, and increased fecundity rates. A high r_m_ value indicates a higher susceptibility of a host plant to insect feeding. In contrast, a low value, as evidenced in maize (0.10 ± 0.0013), suggests that the host plant species possess some level of resistance or tolerance to the pest. Consequently, our findings underscore the substantial growth potential of *S. exigua* under favorable conditions. In their examination of life table parameters of this pest across various host plants, Greenberg et al.^21^ observed that the r_m_ value was highest on pigweed (0.264) and lowest on cabbage (0.156). Discrepancies may arise due to physiological variations among host plant cultivars, genetic disparities from laboratory rearing, or geographic variability within pest populations.

Furthermore, our results suggest that maize is a less hospitable host for *S. exigua* than those examined by Greenberg et al.^21^. Our study unveiled that the highest net reproductive rate (R_0_) of *S. exigua* was documented on chickpea (846.39 ± 18.22), while the lowest R_0_ value was observed on maize (59.50 ± 2.06). Greenberg et al.^21^ reported slightly lower net reproductive rates for *S. exigua*, ranging from 139.3 to 596.0 on cabbage and pigweed. Similarly, within the range of 64.53 to 377.73, the lowest R_0_ value was recorded on various sorghum varieties. Hence, our findings suggest that the net reproductive rate of *S. exigua* in chickpea surpasses previous reports by other researchers. Maize displayed the lengthiest generation time (T) at 42.16 ± 0.129 days, whereas chickpea exhibited the shortest at 29.88 days. Naseri et al.^35^ explored the reproductive performance of *Helicoverpa armigera* (Hübner) (Lepidoptera: Noctuidae) and reported findings akin to ours^28^. Population doubling on sunflower occurred every 3.56 days, compared to 7.22 days on maize. Host suitability is influenced by various factors, including nutrient content, secondary substances, and the insect's digestive and assimilative capabilities^36,37^. To deepen our understanding of insect-plant interactions, fundamental biochemical studies are essential for isolating and identifying phytochemicals that impede the proliferation of *S. exigua* populations on off-season hosts. This research may aid in elucidating pest population dynamics on different host cultivars, facilitating the implementation of management strategies to maintain pest populations below economic injury thresholds.

## Conclusions

Our research offers valuable insights into the suitability of various host plants for the development, longevity, and survival of *S. exigua*. Chickpea and sunflower emerged as the most favorable host plants, providing optimal food quality, followed by castor, blackgram, greengram, groundnut, cotton, and maize. The observed differences in the developmental pattern of *S. exigua* across these resources likely stem from their distinct nutritional profiles and metabolite compositions, impacting the insects' physiology. Our findings suggest that strategies like intercropping, mixed cropping, or crop sequencing with less suitable hosts could influence *S. exigua* population dynamics and reduce its prevalence. Nevertheless, further field-based research is essential to deepen our understanding of this pest's adaptation to diverse environments and host plants, facilitating the development of effective management practices.

## Methods

### Incidence of *S. exigua* on different crops and weeds

Field surveys were conducted in eleven mandals of the Prakasam district, i.e., Darsi, Kurichedu, Addanki, Tallur, Mundlamuru, Markapuram, Pedaraveedu, Cumbum, Chirala, J. Panguluru and Marturu during the *kharif* and post rainy season. During the survey, the incidence of *S. exigua* on different crops and weeds was recorded randomly in each mandal in three villages. Each sample estimate was based on five locations (fields) in each village, and at each location, 25 plants were randomly selected for each cultivated crop. Each location was treated as one replication, and there were fifteen replicates for each sample (five locations and three villages) in each mandal. During the survey, sampling was also done from weed plants available nearby or within the cultivated crop fields. Data was recorded on several larvae of *S. exigua* on 25 randomly selected plants and the extent of foliage damage on a 1–9 scale (1 ≤ 10% leaf area damaged and 9 ≥ 80% leaf area damaged). The observations were recorded during the vegetative, flowering, and pod formation stages in their respective crops (Supplementary Tables 1 and 2). After suitable transformations, the data was subjected to analysis of variance and compared based on the least significant difference (LSD) at *P* = *0.05*.

### Maintenance of stock culture and studies on biology of *S. exigua*

To initiate the laboratory culture, *S. exigua* larvae collected from the field were utilized and reared on their respective host plants in a glasshouse for host conditioning. The lifecycle and fecundity of *S. exigua* were investigated using various host plants, including Chickpea (var. NBeG 3), Sunflower (private hybrid), Blackgram (*var*. LBG 752), Greengram (var. ML 267), Maize (Hy. DHM 117), Castor (*var*. PCH 111), Cotton (var. NDLH 1938), Groundnut (*var*. TAG 24), and Chilli (*var*. Tejaswini). The laboratory experiments were conducted at the Agricultural Research Station in Darsi, Prakasam district, with the assistance of Acharya N. G. Ranga Agricultural University under controlled conditions (27 ± 1 °C and 70 ± 2% RH).

The study involved observing the duration of different developmental stages of *S. exigua* from egg to adult in a laboratory setting. Adults emerging from the respective hosts were placed in a mating chamber (20 cm diameter × 30 cm depth) and provided a 10% honey solution for feeding. Freshly laid egg masses were collected, and each cohort of eggs was placed in labelled plastic Petri dishes (10 cm diameter × 1 cm depth) with a hole in the top cover covered with aluminium wire mesh for aeration. To assess the suitability of different host plants, each host plant was treated as a treatment and replicated 50 times in a randomized design. Upon hatching, each newly emerged larva was individually transferred to a plastic container (9.5 cm top diameter × 6.5 cm bottom diameter × and 12.5 cm high) with a 4 cm diameter ventilated hole in the lid covered with muslin cloth. The larva was fed fresh leaves from each tested host plant until pupation. Moulting was determined by observing the detached head capsule from the newly moulted larva, which was preserved in 70% alcohol to determine the number of instars. After the final moulting stage, the container was filled with vermiculite for pupation, with each larva considered a replication.

The number of days taken to complete each instar was recorded for each cole crop. Other biological and reproductive parameters, such as larval duration, pupal duration, pupal weight, percent pupation, adult emergence, oviposition, pre- and post-oviposition periods, and fecundity, were recorded daily. After adult emergence, pairs of male and female moths (with 20–25 replications) were released in an oviposition chamber covered with fine mesh netting for ventilation. The moths were provided with a 10% honey solution, and test plants were excised and placed in disposable cups at the 3–4 leaf stage as an oviposition substrate to record daily fecundity. The growth index (GI) was calculated by dividing the survival rate of the immature stage by the development time^2^.

### Statistical analysis

The biological and reproductive data were analyzed by one-way analysis of variance (ANOVA), followed by a comparison of the means with least significant difference (LSD) test at α = 0.05 using the online statistical software WASP—Web Agri Stat Package 2.0. The life tables of both the pests were constructed by using ‘TWOSEX-MS Chart’ software. According to the age-stage, two-sex life table principle^38,39^ and method^40^, the following parameters viz., Age-stage-specific survival rates (*S*_*xj*_): ${\text{S}}_{{{\text{xj}}}} = \frac{{{\text{n}}_{xj} }}{{n_{01} }}$, age-specific survival rate (*l*_*x*_): $l_{x} = \sum\nolimits_{j = 1}^{m} {{\text{S}}_{{{\text{xj}}}} }$, age-stage-specific fecundity (*f*_*xj*_), age-specific fecundity (*m*_*x*_): $m_{x} = \frac{{\sum\nolimits_{j = 1}^{m} {S_{xj} f_{xj} } }}{{\sum\nolimits_{j = 1}^{m} {S_{xj} } }}$, age-specific maternity (*l*_x_*m_*x*_), age-stage-specific life expectancy (e_xj_): $e_{xj} = \sum\nolimits_{i = x}^{\infty } {\sum\nolimits_{y = j}^{m} {S^{\prime}_{iy} } }$ age-stage-specific reproductive value (V_xj_): ${\text{V}}_{{{\text{xj}}}} = \frac{{e^{r(x + 1)} }}{{{\text{S}}_{{{\text{xj}}}} }}\sum\nolimits_{i = x}^{\infty } {e^{ - r(i + 1)} \sum\nolimits_{y = j}^{m} {S^{\prime}_{{{\text{iy}}}} {\text{f}}_{{{\text{iy}}}} } }$, intrinsic rate of increase (r): $\sum\nolimits_{x = 0}^{\infty } {e^{ - r(x + 1)} } l_{x} m_{x} = 1$, finite rate of increase (λ): $\lambda = e^{r}$, net reproductive rate (R_0_): $R_{0} = \sum\nolimits_{x = 0}^{\infty } {l_{x} m_{x} }$, mean generation time (T): ${\text{T}} = \frac{{\ln {\text{R}}_{0} }}{r}$ were calculated.

The raw life-history data for *S. exigua* obtained for each of the hosts were entered separately into the notepad (text document). TWOSEX-MS Chart 2024 software (available from the webpage http://140.120.197.173/Ecology/prod02.htm) was used to calculate each population parameter. The means, standard errors and variances of the population parameters by bootstrapping technique (100,000 repetitions). The software greatly simplifies the normally laborious and time-consuming process of calculating all the relevant population parameters. Sigma plot 14.5 was used to create graphs.

### Supplementary Information

Supplementary Tables.

## Data Availability

All data generated or analyzed during this study are included in this article and its supplementary information files. All the figures are original and were prepared by us.
