# Supplementary material for: Off-season survival and life history of beet armyworm, Spodoptera exigua (Hubner) on various host plants
Source: Sci Rep. 2024 Jun 14;14:13721. doi: 10.1038/s41598-024-64639-8 (PMC11178929; doi:10.1038/s41598-024-64639-8)
Supplement: Supplementary file 1 — Supplementary Tables. [file 41598_2024_64639_MOESM1_ESM.docx]

**Supplementary Table 1. Coordinates of beet armyworm surveyed locations on different host crops.**

| Month of survey | Mandal | Lat and long | Name of the crop & Variety | Crop stage |
| --- | --- | --- | --- | --- |
| August, 2016 | Pedaaraveedu | 15.8138° N, 79.2240° E | Onion | Bulb formation |
| August, 2016 | Darsi | 15.7700° N, 79.6794° E | Greengram,  ML 267 | Pod formation |
| September, 2016 | Markapur | 15.7361° N,  79.2691° E | Chilli,  Indam 5 | Vegetative |
| September, 2016 | Chirala | 15.8167° N, 80.3587° E | Groundnut  TAG 24 | Peg formation |
| September, 2016 | Cumbum | 15.5840° N,  79.1098° E | Tobacco nursery | Nursery |
| September, 2016 | Kurichedu | 15.9513° N, 79.4992° E | Castor  PCH 111 | Capsule formation |
| October, 2016 | Martur | 15.9960° N,  80.1034° E | Cotton,  NDLH 1938 | Boll formation |
| October, 2016 | Markapur | 15.7384° N,  79.2805° E | Cow pea, Meghana | Vegetative |
| October, 2016 | J. Panguluru | 15.8046° N, 80.0214° E | Black gram,  LBG 752 | Flowering |
| November, 2016 | Darsi | 15.7700° N, 79.6794° E | Soy bean, JS 335 | Vegetative |
| November, 2016 | Addanki | 15.8159° N,  79.9715° E | Maize,  30V92 | Seedling |
| March, 2017 | Darsi | 15.7548° N, 79.7386° E | Pig weed, *Amaranthus* spp | Flowering |
| March, 2017 | Talluru | 15.7323° N, 79.7687° E | Brinjal,  Mahyco hybrid | Vegetative |
| March, 2017 | Mundlamur | 15.8099° N, 79.9088° E | Sunflower,  Private Hybrid | Heading |
| April, 2017 | Darsi | 15.4024° N,  79.5074° E | *Euphorbia geniculata* | Flowering |

**Supplementary Table 2. Cropping percentage in the mandals surveyed during the survey period**

| **Name of Mandal** | **1** | **2** | **3** | **4** | **5** | **6** | **7** | **8** | **9** | **10** | **11** | **12** | **13** | **14** | **15** | **16** | **17** | **18** | **19** | **20** | **21** | **22** |
| --- | --- | --- | --- | --- | --- | --- | --- | --- | --- | --- | --- | --- | --- | --- | --- | --- | --- | --- | --- | --- | --- | --- |
| Pedaaraveedu | 21.25 | 0 | 0 | 4 | 14 | 1.75 | 0 | 0 | 0 | 0 | 23.5 | 0 | 0 | 0 | 0 | 0 | 19.5 | 8 | 8 | 0 | 0 | 0 |
| Darsi | 31 | 0 | 3.5 | 0 | 8 | 0 | 0 | 8.5 | 0 | 0 | 28 | 0 | 0 | 0 | 0 | 0 | 0 | 5.5 | 15.5 | 0 | 0 | 0 |
| Markapur | 10.25 | 0 | 4.75 | 5.5 | 10.75 | 2.5 | 0 | 0 | 0 | 0 | 29.5 | 0 | 0 | 0 | 0 | 0 | 2.75 | 6.5 | 22.75 | 0 | 0 | 0 |
| Chirala | 36.25 | 3.25 | 0 | 6.5 | 4.25 | 0 | 0 | 0 | 0 | 0 | 10.5 | 0 | 0 | 21.75 | 0.5 | 0 | 0 | 0 | 4.5 | 11 | 1.5 | 0 |
| Cumbum | 31.5 | 6.5 | 5 | 0 | 0 | 8 | 0 | 0 | 3.5 | 0 | 25.75 | 0 | 3.25 | 0 | 0 | 0 | 0 | 11 | 5.5 | 0 | 0 | 0 |
| Kurichedu | 29.75 | 4 | 5.5 | 7.75 | 11.25 | 0 | 0 | 7.5 | 1.25 | 0 | 12.5 | 14.5 | 0 | 0 | 0 | 0 | 0 | 0 | 6 | 0 | 0 | 0 |
| Martur | 16.25 | 1.25 | 0 | 14.75 | 19.5 | 0 | 0 | 0 | 9.75 | 0 | 15.5 | 0 | 0 | 0 | 9.75 | 0 | 0 | 0 | 13.25 | 0 | 0 | 0 |
| Markapur | 10.25 | 0 | 4.75 | 5.5 | 10.75 | 2.5 | 0 | 0 | 0 | 0 | 29.5 | 0 | 4.75 | 0 | 0 | 0 | 2.75 | 6.5 | 22.75 | 0 | 0 | 0 |
| J. Panguluru | 22.25 | 0 | 0 | 10.25 | 16 | 0 | 0 | 4 | 18.75 | 0 | 17.75 | 0 | 0 | 0 | 0 | 0 | 0 | 0 | 11 | 0 | 0 | 0 |
| Darsi | 28 | 0 | 0 | 0 | 8 | 0 | 0 | 0 | 0 | 0 | 28 | 0 | 0 | 0 | 0 | 0 | 0 | 5.5 | 15.5 | 0 | 15 | 0 |
| Addanki | 35.5 | 0 | 4.5 | 22.75 | 18.5 | 0 | 0 | 0 | 0 | 0 | 8.5 |  |  | 0 | 0 | 0 | 0 | 0 | 10.25 | 0 | 0 | 0 |
| Darsi | 18 | 0 | 0 | 0 | 8 | 0 | 0 | 0 | 0 | 0 | 22 | 0 | 0 | 0 | 0 | 0 | 0 | 0 | 0 | 0 | 0 | 0 |
| Talluru | 22.5 | 0 | 0 | 0 | 0 | 0 | 0 | 0 | 0 | 0 | 10.75 | 0 | 0 | 0 | 0 | 0 | 0 | 0 | 0 | 0 | 0 | 9.5 |
| Mundlamur | 14.25 | 0 | 0 | 0 | 0 | 0 | 0 | 0 | 0 | 0 | 8.5 | 0 | 0 | 0 | 8.5 | 10.75 | 0 | 0 | 0 | 0 | 0 | 0 |
| Darsi | 0 | 0 | 0 | 0 | 8 | 0 | 0 | 0 | 0 | 0 | 13 | 0 | 0 | 0 | 18.5 | 0 | 0 | 0 | 0 | 0 | 0 | 0 |

**1-Paddy, 2-Jowar, 3-Bajra, 4-Maize, 5-Cotton, 6-Ragi, 7-Horse gram, 8- Green gram, 9- Black gram, 10-Bengal gram, 11-Redgram, 12-Castor, 13-Cowpea, 14-Groundnut, 15-Sesame, 16- Sunflower, 17-Onion, 18- Tobacco, 19-Chilli, 20-Jute, 21-Soybean, 22-Brinjal.**
